# Regulation of the human tyrosinase gene in retinal pigment epithelium cells: the significance of transcription factor orthodenticle homeobox 2 and its polymorphic binding site

**Published:** 2012-01-10

**Authors:** Mika Reinisalo, Jaana Putula, Eliisa Mannermaa, Arto Urtti, Paavo Honkakoski

**Affiliations:** 1 School of Pharmacy and Biocenter Kuopio, University of Eastern Finland, Finland; 2 Centre for Drug Research, University of Helsinki, Finland

## Abstract

**Purpose:**

Tyrosinase is the rate-limiting enzyme responsible for melanin biosynthesis in the retinal pigment epithelium (RPE) of the eye. Melanin has an important role in retinal development, function, and protection against light-induced oxidative stress, and melanin levels are associated with age-related macular degeneration (AMD). Because the levels of and protection afforded by melanin seem to decline with increasing age, proper regulation of the human tyrosinase gene (*TYR*) in the RPE is an important but insufficiently understood process. Our purpose was to obtain detailed information on regulation of the *TYR* gene promoter in the human RPE and to specify the role of orthodenticle homeobox 2 (OTX2) and microphthalmia-associated transcription factor (MITF).

**Methods:**

We used luciferase reporter constructs to study regulation of the human *TYR* gene promoter in cultured human RPE cells. We further studied the role of OTX2 and MITF, their binding sites, and endogenous expression by using mutagenesis, electrophoretic mobility shift assay, yeast two-hybrid assay, RNA interference, and gene expression analyses.

**Results:**

In the RPE, OTX2 activated the human *TYR* gene promoter via direct trans-activation of novel OTX2 binding elements. In addition, we found that indirect activation by OTX2 via more proximal MITF binding sites, even in the absence of OTX2 sites, took place. These results are consistent with the physical interaction observed between OTX2 and MITF. Overexpression or knockdown of OTX2 in RPE cells resulted in corresponding changes in tyrosinase mRNA expression. Finally, we found that a single nucleotide polymorphism (SNP rs4547091) at the most proximal OTX2 binding site is associated with altered nuclear protein binding and a remarkable decrease in *TYR* promoter activity in RPE cells. This single nucleotide polymorphism (SNP) is more common in the European population in which AMD is also more prevalent.

**Conclusions:**

In the RPE, OTX2 activates the human *TYR* gene promoter by direct DNA binding and by interaction with MITF. Such synergistic interaction highlights the role of OTX2 as a potential coregulator of numerous MITF target genes in the eye. Genetic differences in OTX2 binding sites affect tyrosinase regulation. Collectively, these findings emphasize the role of OTX2 in regulating the human *TYR* gene, with implications for inter-individual differences in melanin synthesis, retinal development, and function as well as susceptibility to retinal degeneration associated with aging.

## Introduction

Tyrosinase (EC 1.14.18.1) is the rate-limiting enzyme involved in melanin biosynthesis, catalyzing dihydroxyphenylalanine (L-DOPA) formation from L-tyrosine. Upon assistance by tyrosinase-related proteins 1 and 2 (TRP-1 and TRP-2), L-DOPA is converted into melanin [1] in pigmented cells such as melanocytes in the skin and retinal pigment epithelium (RPE) cells of the eye [2,3]. Melanin can exert a protective function in tyrosinase-expressing cells in several ways. First, melanin shields these cells from the damage induced by sunlight and ultraviolet radiation [4–6]. Second, melanin may counteract the oxidative stress caused by free radicals derived from lipid peroxidation products [7] and accumulated iron [8,9] in the RPE and in substantia nigra. Such prooxidants may contribute to age-related degeneration of these tissues [10]. Third, the high binding capacity of melanin for metal ions [11] and exogenous chemicals [12–14] also lends support for a protective role of melanin in the eye. In concordance with these findings, melanin [15] and its precursors are essential for the proper development of the retina in mammals [16,17]. Malfunctions in normal expression of tyrosinase [1,16], its post-translational modification [18], or trafficking into melanosomes [19,20] can decrease pigmentation, the stability of the melanosomes [21], and the normal functions of the RPE. Researchers have shown that the content of the RPE cells declines with age [22], perhaps in part due to oxidative degradation [5]. In addition, several age-related changes occur in melanin [22,23], contributing to its functional decline [24].

Despite the importance of proper tyrosinase expression for the RPE, current information on human *TYR* gene regulation has mostly been gained in melanocytes of neural crest origin [25]. Skin melanocytes continuously synthesize and transport melanin into keratinocytes, while the majority of the melanin pigment in the RPE [21] is synthesized prenatally during a few weeks of embryonic development and stored in the melanosomes for the lifespan of the individual. This suggests that any changes in *TYR* gene activity might have profound and long-lasting effects on RPE physiology and health. However, knowledge of tyrosinase expression in the RPE, derived from the neuroectoderm, is scant and based mainly on rodent data [26,27]. Unfortunately, no RPE cell lines display tyrosinase activity and active melanogenesis [28]. Primary RPE cells revert to the depigmented phenotype, their melanosomes disappear, and the ability to form new melanin is lost quickly after cell isolation [2,28,29]. Similar dedifferentiation to the amelanotic phenotype appears in melanoma cells where tyrosinase is not properly sorted into melanosomes but is retained in the endoplasmic reticulum and thereafter degraded [30,31].

The current evidence indicates that two transcription factors, the microphthalmia-associated transcription factor (MITF) and the orthodenticle homeobox 2 (OTX2), are responsible for the correct development of the retina as well as for regulation of melanogenic genes. MITF’s importance is highlighted by certain human *MITF* [32] and mouse *Mitf* [33]gene mutations that result in abnormal melanogenesis, compromised RPE function, and visual problems. Researchers have shown that binding MITF to proximal binding sites (termed *M* and *E boxes*) and to a more distal DNA element (termed *TDE*) is important for the expression of the *TYR* gene in melanocytes [25,34,35]. Isoforms of MITF that differ in their N-terminal domains due to alternative promoter usage [36,37] are expressed in a cell-specific manner: the MITF-M isoform is present in the melanocytes while the dominant isoforms in the mouse RPE are A, D, H, and J. Isoforms A and J are also present in the neural retina [37,38]. However, very little is known about their roles with respect to *TYR* gene regulation: the reporter gene activity is retained even with N-terminally truncated MITF variants [37]. Therefore, we focused here on the best-characterized MITF-A isoform.

The homeodomain protein OTX2 is involved in the development of the retina [39,40] and brain [41–43], and mutations in the human *OTX2* and mouse *Otx2* genes have been associated with severe ocular malformations [44–46]. OTX2 appears to regulate enzymes necessary for the function of the neural retina and the RPE such as the human interphotoreceptor retinoid binding protein [47] and the human DOPAchrome tautomerase involved in melanin synthesis [48]. More recently, OTX2 has been found to activate the mouse *Tyr* [49] and human bestrophin-1 (*BEST1*) gene promoters [50]. However, detailed studies on regulation of the human *TYR* in the RPE are not clear, and the role of the OTX2 factor has not been studied with this gene promoter. This scant knowledge is associated with the scarcity of good RPE cell models and lack of efficient gene delivery methods [51]. Moreover, dissimilarities between the rodent and human *TYR* promoter sequences near and at putative OTX2 binding sites (ClustalW analysis of promoters up to −462 bp) may compromise the use of mouse models. Therefore, our aim was to obtain more detailed information on regulation of the human *TYR* promoter and mRNA (mRNA) in human RPE cells by using the reverse transfection technique [52] that enables analysis of weak gene promoters. Due to the lack of cells capable of melanogenesis, we used two well established human RPE cell lines (ARPE-19 and D407) differing in tyrosinase mRNA expression.

## Methods

### Chemicals

Cell culture media, fetal bovine serum (FBS), and supplements were either from Invitrogen Corporation (Gaithersburg, MD) or Sigma (St. Louis, MO). The molecular biology reagents were from Finnzymes (Espoo, Finland), MBI Fermentas (Vilnius, Lithuania), GE Healthcare (Uppsala, Sweden), or Promega (Madison, WI). Polyethyleneimine 25 (PEI25) and other reagents of the highest grade available were from Sigma.

### Retinal pigment epithelium and melanoma cell lines

RPE cell lines ARPE-19 (CRL-2302; ATCC, Rockville, MD) and D407 [53] were maintained as previously described with media and supplements from Invitrogen [51]. G-361 melanoma cells (CRL-1424, ATCC) were maintained in McCoy’s 5A medium (M8403, Sigma) supplemented with 10% FBS, 2 mM L-glutamine, 100 U/ml penicillin, and 100 μg/ml streptomycin. The ARPE-19 cells were grown at 37 °C and 7% CO_2_, whereas the other cells were kept at 37 °C and 5% CO_2_. Prolonged culture of G-361 or D407 cells beyond 96 h was not possible due to cell detachment.

### Bovine and fetal human primary retinal pigment epithelium cells

Bovine RPE cells (bRPE) were isolated [54] with minor modifications. Briefly, bovine eyes were collected from a local slaughterhouse. The first anterior segment, vitreous, and neural retina were removed. Eyecup segments were quickly washed with 1 ml 0.05% trypsin/0.5 mM EDTA (EDTA) solution. Fresh trypsin/EDTA (1 ml) was added and incubated for 15 min. The detached cells were transferred into a tube containing culture medium (DMEM-F12 [Gibco Invitrogen] supplemented with 10% FBS, 2 mM L-glutamine, 100 U/ml penicillin, and 100 μg/ml streptomycin) and centrifuged at 390× g for 5 min. After centrifugation, the cell pellet was suspended in 3 ml of fresh culture medium and plated onto 6-well plates. After 3 h, the culture medium was replaced. Cells were maintained at 37 °C and 5% CO_2_ in the culture medium with two medium changes per week. Upon inspection, primary cells were polygonal containing many heavily pigmented, oval melanosomes that were lost during passaging. Their pigment cell origin was confirmed by the expression of TRP-1 (data not shown). Fetal human primary RPE cells (fhRPE) have been described [55,56]. The frozen cells were thawed, and the cells were maintained at 37 °C and 5% CO_2_ in DMEM medium supplemented with 15% FBS (Invitrogen) and 2 mM L-glutamine, 100 U/ml penicillin, and 100 μg/ml streptomycin, 50 µg/ml gentamicin, and 1 ng/ml bFGF (Sigma).

### RNA isolation and quantitative reverse transcription polymerase chain reaction

Continuous human cell lines were seeded at high density on 6-well plates (1×10^6^ cells per well) to reach confluency by 48 h, and total RNA was isolated by using TRI Reagent (Sigma). Isolated RNA was treated with DNase (DNA-Free Kit, Ambion, Austin, TX). The RNA concentration was determined with RiboGreen quantitation reagent (Molecular Probes, Eugene, OR). Synthesis of cDNA (cDNA) was performed by using M-MuLV reverse transcriptase (Fermentas) as reported [51]. Intron-spanning primers for human *TYR* mRNA (forward 5′-AGC ACC CCA CA AAT CCT AAC TTA C-3′ and reverse 5′-ATG GCT GTT GTA CTC CTC CAA TC-3′), *MITF-D* mRNA (5′-TTT TAA CCT GAC AGG CTT TGA ATA CAG-3′ and 5′-CAA GAT GCG TGA TGT CAT ACT GG-3′), and *MITF-H* (5′-TTC AGA TGT TCA TGC CAT GCT C-3′ and 5′-GCG TAG CAA GAT GCG TGA T G-3′) were designed by using Primer Express (Applied Biosystems, Foster City, CA), OLIGO (Molecular Biology Insights, Inc., Cascade, CO) and BLAST programs. TaqMan primer/probe sets for *MITF-A* (Hs01115553_m1), *MITF-M* (Hs00165156_m1), and *OTX2* (Hs00222238_m1) were ordered from Applied Biosystems. Quantitation of *TYR*, *MITF-D*, and *MITF-H* mRNAs was performed with the SYBR Green PCR Master Mix (Applied Biosystems) with final primer concentrations of 100 nM. Sequence-specific amplification of cDNAs was verified by performing melting-point analysis. For SYBR Green PCR as well as TaqMan PCR (TaqMan Universal PCR Master Mix, Applied Biosystems), 40 ng of cDNA was used in the final 15 µl reaction volume. All quantitative PCRs were performed on an ABI Prism 7500 instrument (Applied Biosystems). Because the β-actin (*ACTB*) mRNA increased during the long-term maintenance of the APRE-19 monolayer (data not shown), the threshold cycles (Ct) obtained were normalized with RNA amounts in cDNA synthesis, as instructed by Bustin [57].

For the fhRPE cells, the RNA was isolated and quantified with the TaqMan Gene Expression Cells-to-CT Kit (Applied Biosystems). The threshold cycles (Ct) were normalized to endogenous β-actin mRNA expression. The TaqMan primer/probe set for the human *ACTB* mRNA (forward 5′-CGG CAC CAC CAT GTA CCC-3′ and reverse 5′-ACA CGG AGT ACT TGC GCT CA-3′; probe 5′-VIC-ATC AAG ATC ATT GCT CCT C-MGB-3′) was designed using Primer Express (Applied Biosystems, Foster City, CA). Data analysis was performed with the Q-Gene program (Equation 2) [58].

### Construction of the human *TYR* gene promoter and mutagenesis

The human *TYR* promoter fragments were amplified with high-fidelity PCR from human genomic DNA, and all constructs were verified with dideoxy sequencing. Details of the cloning are listed in Appendix 1. The established MITF and putative OTX2 binding sites were mutated by using the QuickChange II XL Site-Directed Mutagenesis Kit (Stratagene, La Jolla, CA) according to the manufacturer’s instructions with designed mutagenic primers (Appendix 2). The mutated promoter constructs were identified with diagnostic restriction enzyme digestions and verified with dideoxy sequencing.

### Reverse transfection of tyrosinase promoter constructs

To assess the functionality of the cloned promoters, reporter constructs were delivered into cells by using reverse transfection on 48-well plates essentially as described [52]. Reverse transfection is an efficient gene delivery method for hard-to-transfect cells such as primary cells of the retina wherein carrier/DNA complexes are freeze-dried on the surface of culture plates and stored for an extended time until used for transfection. In preliminary experiments, the PEI25/DNA charge ratio of +5 was found to give optimal transfection efficiency and minimal cell toxicity for all cell types. The PEI25/DNA complexes contained 0.5 µg *TYR* promoter-driven reporter, 0.5 µg *MITF-A* [38], or *OTX2* (Origene Technologies, Inc., Rockville, MD) expression vector, 0.2 µg CMV promoter-driven pCMVβ control reporter, and an empty pCR3 expression vector (Invitrogen) to adjust the total DNA amount to 1.7 µg per well. The reverse transfection plates were prepared and stored in a desiccator without any decrease in transfection efficiency for several months [52] up to one year (unpublished data). The thymidine kinase (*TK*) promoter-driven [51] and promoterless pGL3-Basic (Promega) reporter plasmids were used as controls in every experiment. For transfection experiments on 48-well plates, optimized cell numbers were used (ARPE-19: 1.2×10^5^ and D407: 0.5×10^5^ cells per well). After 48 h, cells were lysed in 70 µl of lysis buffer, and lysates were assayed by using the Victor Multilabel Plate Reader (PerkinElmer, Turku, Finland). Quantitation of luciferase and β-galactosidase (GLB1) was performed as described earlier [48] with 40 µl and 20 µl of lysate, respectively. Reverse transfection of primary fhRPE (0.65×10^5^ cells per well, passages 4–5) and bRPE (0.1×10^5^ cells per well, passages 9–10) cells was performed as above except that the PEI25/DNA complexes were prepared with 1.0 µg of *TYR* promoter-driven reporter and 0.4 µg of pCMVβ. High concentrations of the OTX2 expression vector may activate some viral promoters [50]. We observed approximately 50% activation of the pCMVβ control reporter when cells were transfected with MITF-A or OTX2, and less than approximately 2.7 fold activation with their cotransfection. When the final data was processed, CMV promoter activation was taken into account by normalizing β-galactosidase activities with the activation factor relative to the empty pCR3 vector. In control experiments, β-galactosidase transfection efficiency was stable with less than 15% variation between wells transfected with the same expression constructs. All transfections were done on two or three separate occasions with at least three replicate wells per each condition.

### Preparation of nuclear extracts and electrophoretic mobility shift assays

For electrophoretic mobility shift assays (EMSAs), oligonucleotide probes containing the wild-type and mutated binding sites for MITF and OTX2 were designed (Appendix 3). Double-stranded probes were labeled with [α-^32^P]dCTP (GE Healthcare and PerkinElmer) by using the Klenow fragment (MBI Fermentas) and purified with Quick Spin columns (GE Healthcare). The preparation of nuclear extracts from APRE-19 cells cultured for 14 days and the optimized conditions for the EMSA reactions (20 µl) are detailed in Appendix 1. Intensities of the specific complexes were quantified by using Quantity One software (Bio-Rad, Hercules, CA). Specific binding of OTX2 was demonstrated by performing supershift EMSAs. Nuclear extracts from ARPE-19 cells cultured for 14 days were incubated with the anti-OTX2 antibody [59], or alternatively, the Flag-OTX2 expression vector (RC207479, Origene) or empty pCR3 (5 µg/well) was reverse transfected into the D407 cells on 6-well plates (0.4×10^6^ cells per well), and nuclear extracts were incubated with the anti-Flag antibody (Origene).

### Transient transfection of orthodenticle homeobox 2 into D407 cells

**Figure 1:**
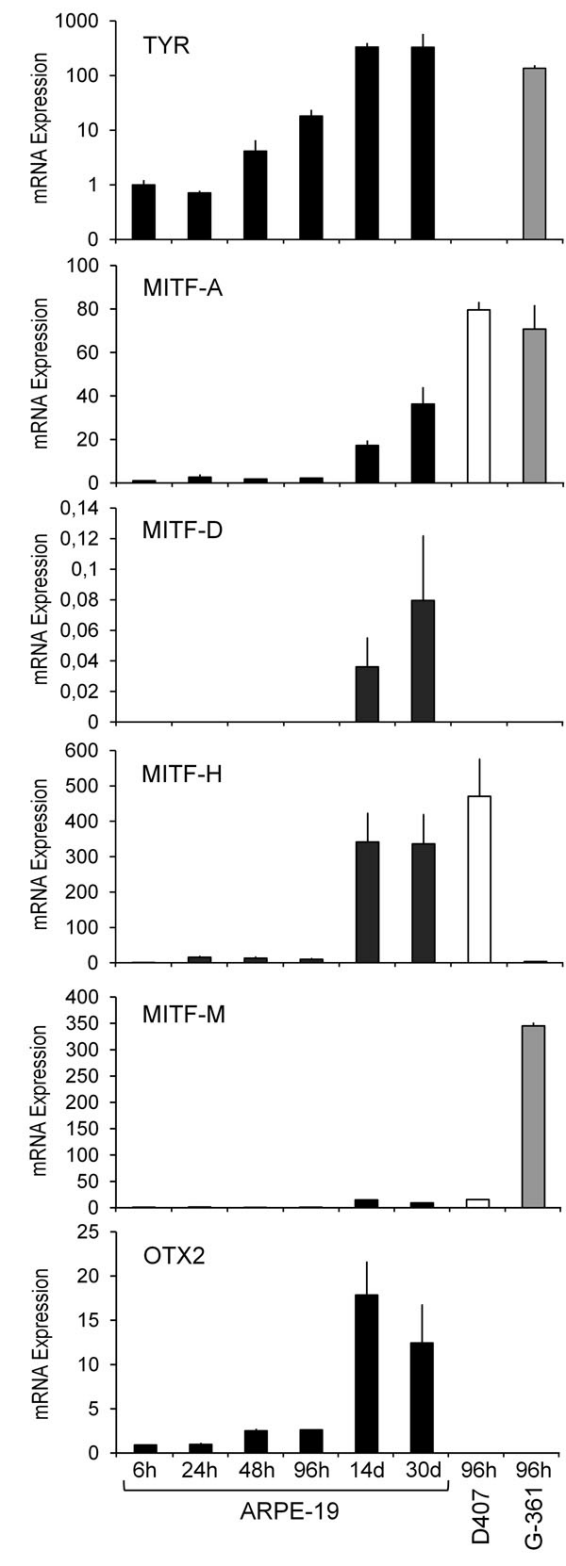
Expression of *TYR*, *MITF-A*, *MITF-D*, *MITF-H*, *MITF-M*, and *OTX2* mRNA in RPE cells. ARPE-19 mRNA was collected at various time points (6 h, 24 h, 48 h, 96 h, 14 days, and 30 days). Other samples were isolated at 96 h from D407 RPE cells and G-361 melanoma cells, a positive control for *TYR* expression. Results are presented as the mean normalized expression±SD relative to 6 h ARPE-19 culture (=1) from three to four independent cultures each performed in triplicate. Due to very low expression levels, *MITF-D* values were normalized relative to *MITF-H* (6 h ARPE-19 culture=1).

The RPE cell line D407 does not appear to express endogenous OTX2 or tyrosinase mRNAs (see Figure 1 [48,50]). The OTX2 expression vector (0–1.5 µg/well) was delivered into the D407 cells as described earlier [51] by using conventional transfection with PEI25 on 48-well plates (0.5×10^5^ cells per well). Isolation of RNA, DNase treatment, and cDNA synthesis were performed 24 h after transfection by using the TaqMan Gene Expression Cells-to-CT Kit. Endogenous tyrosinase mRNA was quantified and normalized to β-actin mRNA expression as described above.

### Silencing of orthodenticle homeobox and microphthalmia-associated transcription factor mRNA expression in ARPE-19 cells with RNA interference

The role of OTX2 and MITF-A as regulators of tyrosinase expression was studied by using OTX2- and MITF-specific Silencer Select small interfering RNAs (siRNAs: s9933, s8790; Ambion) at a final concentration of 25 nM. Shortly, 24 h after plating on 48-well plates (0.75×10^5^ cells per well) the ARPE-19 cells were transfected with the TransIT-TKO Transfection Reagent (Mirus, Madison, WI) according to the manufacturer’s instructions. RNA isolation, DNase treatment, and cDNA synthesis were performed 48 h after transfection by using the TaqMan Gene Expression Cells-to-CT Kit. Silencer Select Negative Control #1 siRNA (Ambion) was used to monitor the effects of siRNA delivery in each experiment. Endogenous *TYR*, *OTX2*, and *MITF-A* mRNAs were quantified and normalized to *ACTB* mRNA expression as described above.

### Yeast two-hybrid assay

To study potential protein–protein interactions between the *OTX2* and *MITF* transcription factors, constructs coding for full-length proteins (or reported functional domains thereof) were created by using the pGBKT7 and pGADT7 plasmids within the yeast two-hybrid system (Matchmaker GAL4 System 3, Clontech Laboratories Inc., Mountain View, CA). Desired sequences were amplified from the OTX2, MITF-A, or MITF-M [34] expression vector by using the high-fidelity Phusion DNA polymerase (Finnzymes) and sequence-specific primers (primer sequences available upon request). Amplified *MITF-A* and *MITF-M* products were digested with NdeI and SalI and cloned into the NdeI/SalI-digested pGBKT7 GAL4 DNA-binding domain vector, resulting in yeast “bait” vectors coding for full-length MITF-A (FL: residues 1−520) or its fragments (A1−AD: residues 1−297, A1−B1b: residues 1−124, B1b−AD: residues 36−297, AD: residues 118−297), or MITF-M (FL: residues 1−419, M1−AD: residues 1−196). The common central and C-terminal domains of MITF-A and MITF-M isoforms were cloned into pGBKT7 vectors by NdeI/SalI digestions to produce fragment constructs (bHLH-LZ: MITF-A residues 295−405, S: MITF-A residues 402−520). Amplified OTX2 products were digested with EcoRI and BglII and cloned into the EcoRI/BglII-digested pGADT7 vector that resulted in yeast “prey” vectors encoding the full-length OTX2 (FL: residues 1−297) or its fragments (ND: residues 1−37, HD: residues 37−109, CD: residues 107−297) fused to the GAL4 activation domain. As a positive control, the established bait/prey interaction between the mouse constitutive androstane receptor ligand-binding domain (mCAR in pGBKT7) and the mouse steroid receptor coactivator-1 peptide (mSRC-1 in pGADT7) was used [60]. The yeast transformations and β-galactosidase assays were performed according to the manufacturers’ instructions.

## Results

### In ARPE-19 cells, levels of tyrosinase and orthodenticle homeobox 2 mRNAs increase in parallel

Amelanotic human ARPE-19 cells are commonly used as a model for pigmented RPE cells [61]. Tyrosinase mRNA was upregulated already at 48 h, reaching more than a 300-fold increase at extended culture for 14–30 days (Figure 1, top panel). These levels exceed those in G-361 melanoma cells while another RPE cell line D407 lacking pigmentation [53] did not express tyrosinase mRNA. ARPE-19 cells can be maintained as a monolayer for extended periods due to the cells’ contact inhibition [61], while for D407 and G-361 cells, cell detachment precluded reliable mRNA quantification in cultures exceeding 96 h. The transcription factor MITF-A mRNA was expressed at remarkably constant levels (≤twofold differences) during the first 96 h after which it increased 17 to 36 fold. The *MITF-A* mRNA was present at even higher levels in tyrosinase-expressing G-361 cells and D407 cells devoid of *TYR* mRNA (second panel). *MITF-D* mRNA was expressed in ARPE-19 cells at very low levels, reaching the highest activity during the extended cultures of 14–30 days (third panel) while it was not expressed in D407 or G-361 cells. In contrast, *MITF-H* mRNA was expressed in both types of RPE cells, shared a similar expression pattern with *MITF-A*, and reached more than a 300 fold increase at 14–30 days (fourth panel). The ARPE-19 cells expressed low levels of *MITF-M* mRNA with 10 to 15-fold increases occurring upon extended culture. *MITF-M* mRNA was also present in D407 cells and at high levels in G-361 cells, as expected (fifth panel). In contrast to the *MITF* factors, the *OTX2* mRNA was upregulated threefold already at 48 h in the ARPE-19 cells, reaching an 18-fold increase at 14 days. The G-361 and D407 cells did not express *OTX2* mRNA (Figure 1, bottom panel). In summary, upregulation of *OTX2* and *TYR* mRNAs precedes the enhanced expression of *MITF* transcripts in ARPE-19 cells, and high levels of MITF-A, MITF-H, and MITF-M are not sufficient to trigger *TYR* expression in D407 cells. These temporal and cell-dependent results prompted us to study the association of *OTX2* and *TYR* gene expression in more detail.

### Activities of the human tyrosinase promoter constructs in retinal pigment epithelium cells

**Figure 2:**
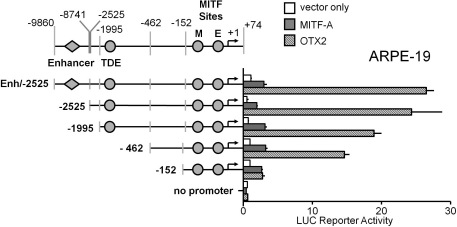
OTX2 activation of the human *TYR* promoter is mediated through the proximal region at −462 to −152. Luc-reporter constructs containing the human *TYR* promoter −2525 with enhancer element −9860 to −8741 (=Enh./-2525), deletions −2525, −1995, −462, −152, and promoterless pGL3-Basic were cotransfected into ARPE-19 cells with MITF-A, OTX2, or an empty expression vector (pCR3). Normalized luciferase activities are relative to the *TYR* promoter construct −152 (=1) cotransfected with the empty expression vector (pCR3). Data are means±standard error of the mean (SEM) from two independent transfections each performed in triplicate.

To determine the DNA elements involved in basal and transcription factor-mediated expression, we cloned several fragments of the human *TYR* promoter (−1995/+74) carrying the established MITF binding sites M box (−104), *E box* (−12), or *TDE* (−1861) and distal regions most homologous to the mouse *TYR* gene (−9860/-8741 and −2525/-1996) upstream of the luciferase reporter (Figure 2, top). We transfected ARPE-19 cells with these reporter constructs together with empty, *MITF-A*, or *OTX2* expression vectors, and cultured them for 48 h. Transfection of either *MITF-A* or *OTX2* resulted in more than 30 fold enhancement of the respective transcription factor mRNA, with no discernible effect on the expression of the reciprocal factor (data not shown). During the DNA transfection period, the endogenous *OTX2* and *TYR* mRNAs displayed a slight increase while the *MITF* mRNAs remained stable (Figure 1). During the same time, the basal reporter activities of the *TYR* promoter in ARPE-19 were low but clearly detectable (Figure 2, white columns), and deletion of the *enhancer* and *TDE* barely decreased the activity. The D407 cells gave similar results (Appendix 4). In the ARPE-19 cells, approximately threefold upregulation by MITF-A of reporter activity required the proximal DNA region −152/+74 that carried M and E boxes (Figure 2, gray columns). In the D407 cells, the activation by MITF-A was significant (>2 fold) with −462 or longer constructs (Appendix 4). In contrast to the modest effects of MITF-A in ARPE-19 cells, OTX2 activated the longer reporter constructs robustly (19 to 26 fold; Figure 2, hatched columns). The bulk of the OTX2-dependent activity resided in the fragment −462/-153, and the proximal promoter −152/+74 still mediated approximately threefold activation. The results with the D407 cells were rather similar to those of the ARPE-19 cells (Appendix 4). Thus, OTX2 can upregulate the *TYR* gene promoter in RPE cells to a much higher degree than MITF-A.

### The relative importance of microphthalmia-associated transcription factor-A and orthodenticle homeobox 2 binding sites in the human tyrosinase proximal promoter

**Figure 3:**
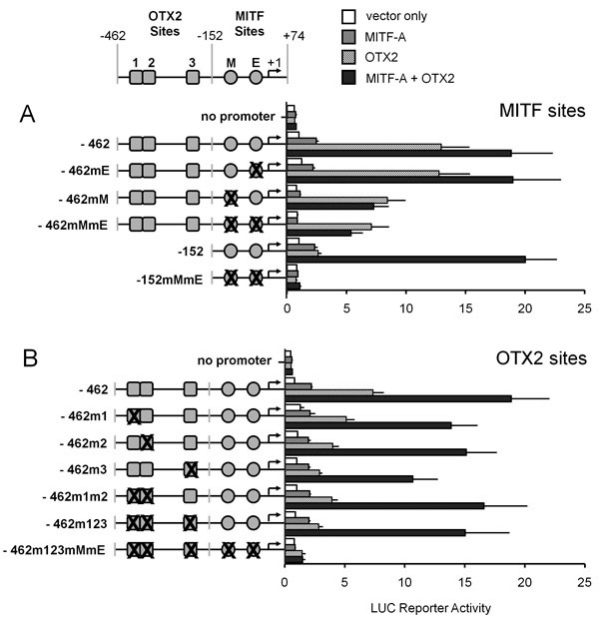
OTX2 can activate the human *TYR* promoter even in the absence of DNA binding sites. OTX2 directly activates the *TYR* promoter through specific OTX2 binding *sites 1* (−361 to −356), *2* (−354 to −349) and *3* (−226 to −221) and indirectly via MITF sites. **A**: Luc-reporter constructs containing disrupted MITF binding sites at positions −104 to −99 (mM=mutation of the *M box*) and −12 to −7 (mE=mutation of the *E box*). **B**: Disruptions of OTX2 binding *sites 1*, *2*, and/or *3* (renamed m1, m2, and m3) in the human *TYR* promoters −152 or −462 were cotransfected into ARPE-19 cells with MITF-A or OTX2 factors, their combination, or with an empty expression vector (pCR3). Normalized luciferase activities are relative to *TYR* promoter construct −152 (=1) cotransfected with the empty expression vector pCR3 (=vector only). Data are means±SEM from two independent transfections each performed in triplicate.

We next focused on the fragment −462/+74, which contained the most important elements for OTX2 and MITF-A responses in ARPE-19 cells. Three motifs similar to the OTX2 binding site TAATCY [62] were found at positions −361, −354, and −226, upstream of the *M* (−102) and *E boxes* (−12; Appendix 2 and Appendix 3). The effects of MITF-A or OTX2 or their combination on these proximal *TYR* promoters carrying either wild-type or mutated MITF binding sites are shown in Figure 3A. Mutagenesis of the M box decreased the basal (white columns) and MITF-A-dependent (gray columns) promoter activities in ARPE-19 cells by approximately 50% while mutation of the E box had no discernible effect. Disruption of MITF sites and the *M box* alone decreased the trans-activation by OTX2 markedly (hatched columns; from 13- to sevenfold), even though OTX2 binding sites 1 to 3 were intact in the −462 construct. MITF-A and OTX2 activated the −152 construct by approximately 2.5 fold, but surprisingly, their cotransfection was synergistic (20 fold; black columns) even in the absence of any OTX2 binding sites. In the D407 cells (Appendix 5), the results recapitulated the effects seen in the ARPE-19 cells, i.e., activation by OTX2 depended partially on the M box, and synergism was observed with the −152 construct. Taken together, the M box is most important for MITF-A’s control of the *TYR* promoter in ARPE-19 cells. Moreover, OTX2 acts directly via OTX2 binding sites and indirectly via MITF sites, preferentially the *M box*.

Next, the putative OTX2 binding sites were mutated and reporter activities analyzed in a similar fashion (Figure 3B). In the ARPE-19 cells, none of the OTX2 site mutations decreased basal or MITF-A-dependent activities (white and gray columns). The OTX2-dependent activity was decreased by all mutations (hatched columns), although most sensitively by mutation at site 3. Mutation of all OTX2 sites (construct −462m123) reduced the OTX2-dependent activity by 65%, and the remaining threefold activation was dependent on the MITF binding sites. The synergistic activation by MITF-A and OTX2 was slightly reduced by OTX2 site mutations, with the largest decrease (45%) seen again with the mutation at site 3. Similar results were obtained with D407 cells (Appendix 6). Thus, binding site 3 is the major determinant of direct OTX2 activation in ARPE-19 cells.

### A functional single nucleotide polymorphism is located in orthodenticle homeobox 2 binding site 3

**Figure 4:**
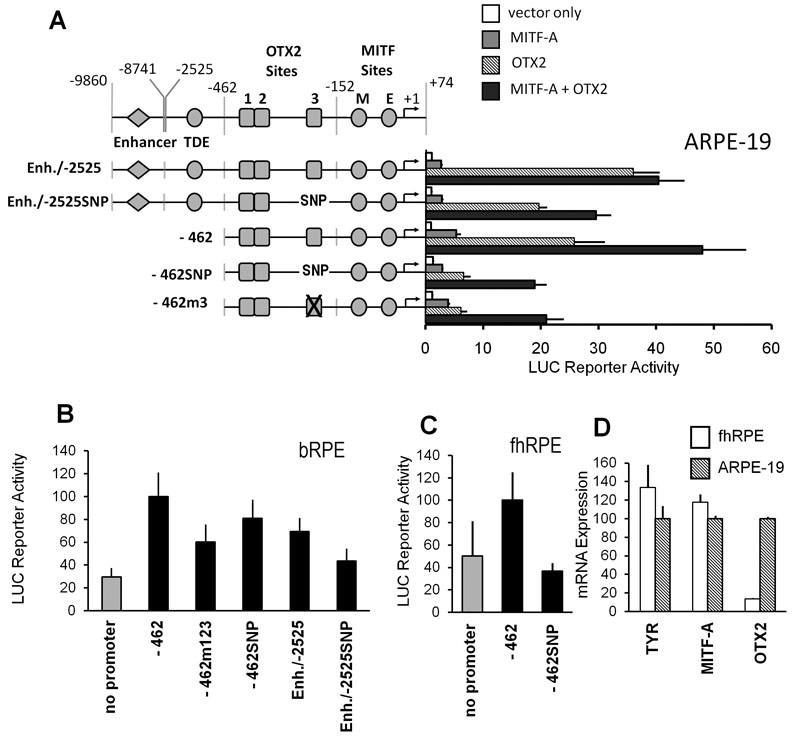
A SNP in the OTX2 binding site 3 decreases the activity of the human *TYR* promoter. **A–C**: The T-223C (rs4547091) mutation (marked here as SNP) was introduced to reporters containing the *TYR* promoter fragments Enh./-2525 and −462. The wild-type and mutated reporters and −462m3 or −462m123 plasmids were transfected into ARPE-19, bovine (bRPE), and human fetal primary RPE (fhRPE) cells with MITF-A or OTX2 factors, their combination, or an empty expression vector (pCR3). Data are means±SEM from two independent transfections performed in triplicate or quadruplicate. **D**: The endogenous expression of tyrosinase, MITF-A, and OTX2 mRNA in fhRPE cells was measured at time point 48 h. Mean normalized expression±SEM are shown relative to 48 h ARPE-19 culture (=100) from two independent cultures each performed in triplicate.

Comparison of the promoter sequence with the National Center for Biologic Information Single Nucleotide Polymorphism database (Table 1) indicated that a single nucleotide polymorphism (SNP; rs4547091, T/C at −223) was located within the OTX2 binding site 3. The frequency of this SNP varies dramatically, with individuals of African ancestry carrying mostly the T/T genotype (73.9%) while individuals of European or Asian ancestry have a preponderance of the C/T (45.5%) or C/C (63.6%) genotype, respectively. The T-223C mutation was introduced into the long and proximal *TYR* promoter constructs (Figure 4A and Appendix 7). We found that this SNP decreased OTX2-dependent activities (hatched columns) by up to 74%, and with the −462 construct, the effect of the SNP was practically equal to the disruption of site 3. The synergistic effect of MITF-A and OTX2 was also decreased by up to 61% (black columns). To study these findings in a more natural setting, we transfected the wild-type and SNP-carrying reporter constructs into primary bovine and fetal human RPE cells. Although the primary cells replicated slower and their transfection efficiencies were lower than for the RPE cell lines, the SNP reduced the reporter activities in primary bovine cells (Figure 4B) and in fetal human primary RPE cells (Figure 4C). The latter cells also expressed endogenous tyrosinase, MITF-A, and OTX2 mRNAs (Figure 4D). In summary, transcription of the *TYR* gene in RPE cells might be affected by genetic differences in the OTX2 binding site 3 among individuals.

**Table 1:** The frequency of SNP rs4547091 in different human populations.

**Population**	**Allele frequencies**		
	C/C	C/T	T/T
African American	0.043	0.217	0.739
European	0.273	0.455	0.273
Asian	0.636	0.273	0.091
**Fold activation by OTX2 in ARPE-19 cells**	**Genotype in construct**		
	C/C		T/T
Enh/-2525 construct	19.7 (55%)		36.0 (100%)
−462 construct	6.7 (26%)		25.8 (100%)

The frequency data for the SNP ID rs4547091 (T to C) at location chromosome 11, position 8855046 were retrieved from the NCBI SNP database (rs4547091). The reporter activity data was derived from Figure 4A.

### Specific nuclear proteins bind to orthodenticle homeobox 2 and microphthalmia-associated transcription factor-A sites in ARPE-19 cells

**Figure 5:**
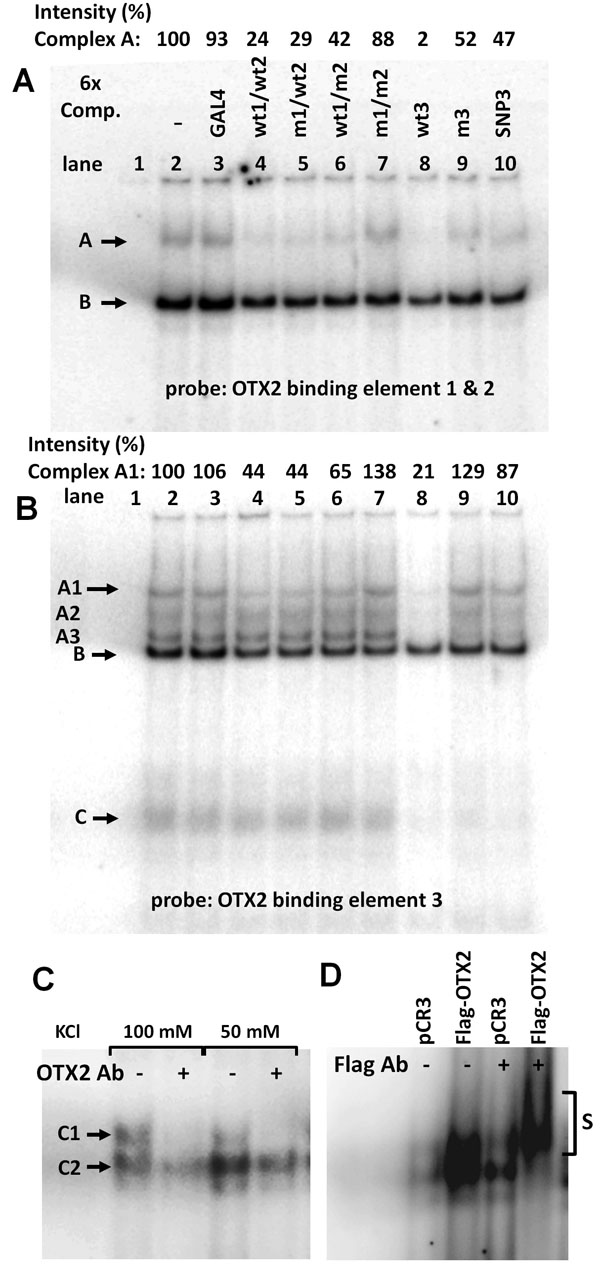
Specific nuclear protein binds to OTX2 binding elements in the human tyrosinase promoter. ARPE-19 cells were cultured for 14 days, and nuclear extracts were prepared. Binding of nuclear proteins to labeled OTX2 probes 1 and 2 (**A**) and 3 (**B**) was competed with only a sixfold excess of unlabeled probes for unrelated GAL4 (lanes 3), wild-type or mutated OTX2 sites 1 and 2 (lanes 4–7), wild-type or mutated OTX2 site 3 (lanes 8–9), and SNP rs4547091 at OTX2 site 3 (lanes 10). Complexes showing specific DNA-nuclear protein interaction are indicated with the symbol A, A1, A2, A3, or C. Non-specific DNA-nuclear protein complexes are indicated with **B**. Free probes in the absence of nuclear extracts are on lanes 1, and full reactions without competitor are on lanes 2. Above the gel images, intensities of the most specific DNA nuclear protein complexes A (**A**) and A1 (**B**) are shown relative to nuclear extracts without competitor (lanes 2=100). **C**: Supershift assay of OTX2 binding site 3 was performed by incubating nuclear extracts from ARPE-19 cells with or without the OTX2 antibody. Strong and modest inhibition of the specific complexes C1 and C2, respectively, was observed. **D**: D407 cells were transfected with the Flag-tagged OTX2 cDNA or with empty control plasmid (pCR3), and nuclear extracts were prepared and incubated with the Flag-specific antibody. The supershifted DNA–protein complex is indicated with the symbol S. Both supershift assays were controlled by using a non-specific control antibody (data not shown).

Human nuclear protein binding to *TYR* promoter elements in RPE cells has never been studied before, and DNA binding has been assessed only with recombinant proteins in vitro [35,49]. Because *TYR* regulation may involve post-translational modification absent in the recombinant MITF and OTX2 proteins, we prepared nuclear extracts from 14-day ARPE-19 cell cultures (when *MITF-A* and *OTX2* mRNAs are expressed and the *TYR* gene is activated), and DNA binding and competition experiments were performed under optimized conditions. Because the OTX2 *sites 1* and *2* are very close to each other, we used a probe covering both sites and observed two main complexes (Figure 5A). The upper complex A was highly specific, because it could be competed by only a sixfold excess of unlabeled probes for OTX2 sites 1 and 2 or site 3. The competition was less efficient by probes containing single mutations at site 1 or 2, and little or no competition was detected by an unrelated probe, the double mutant 1 and 2 probe, and when site 3 was mutated or carried the SNP. The lower complex B was non-specific. We detected a more intricate pattern with the OTX2 probe 3 (Figure 5B). At least three specific, slowly migrating complexes (A1 to A3) were competed by only a sixfold excess of unlabeled OTX2 probe 3 but not when the site 3 was mutated or contained the SNP. Partial competition of the A1 complex was observed with the wild-type probes 1 and 2 but not when these OTX2 sites were mutated. In addition, a diffuse and fast-migrating doublet complex C was competed in a similar fashion to complexes A1–A3. When the specificity of OTX2 binding was studied with the supershift assay (Figure 5C), the formation of complex C was inhibited by the OTX2-specific antibody [59]. When D407 cells were transfected with the Flag-OTX2 plasmid, we found a great increase in the intensity of complex C and a supershift by the anti-Flag antibody (Figure 5D). With the MITF M box and E box probes (Appendix 8), we detected single specific complexes A and A1, respectively, which were competed out only by wild-type but not by mutated M or E box or unrelated probes. Therefore, specific binding proteins for MITF-A and OTX2 sites exist in ARPE-19 cell nuclei. The effects of mutations on DNA binding correlated remarkably well with the cotransfection assays: especially, the SNP at OTX2 site 3 attenuated nuclear protein binding and reporter activation.

### Expression of orthodenticle homeobox 2 activates tyrosinase mRNA expression in D407 cells

**Figure 6:**
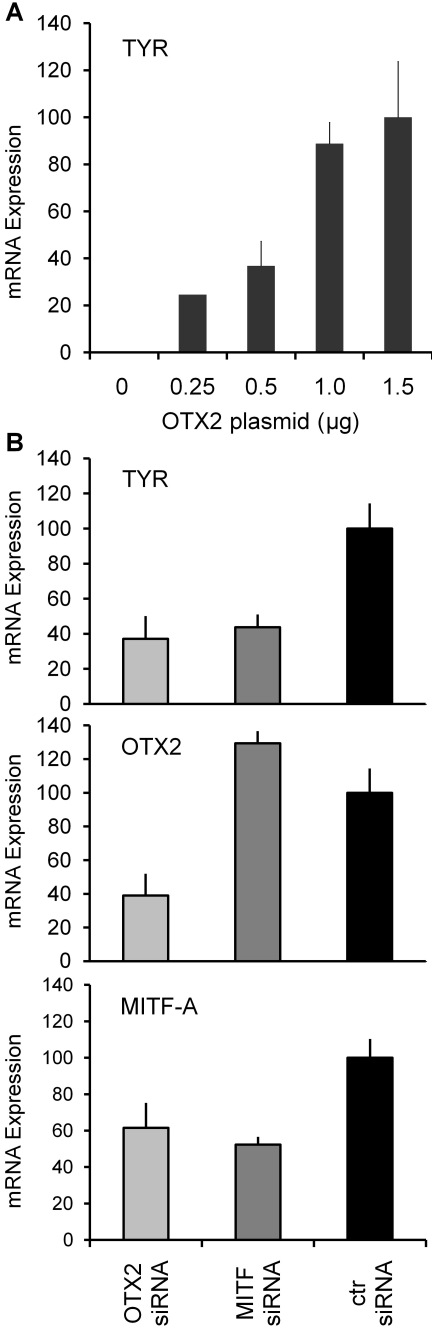
Modulation of OTX2 levels in RPE cells results in parallel expression of tyrosinase mRNA. **A**: Various amounts (0 to 1.5 µg) of OTX2 expression plasmid were transfected into D407 cells followed by measurement of tyrosinase mRNA expression. Results are presented as the mean normalized expression±SEM relative to maximal OTX2 expression plasmid amount (1.5 µg=100) from three independent transfections each performed in triplicate. **B**: Endogenous tyrosinase, OTX2, and MITF-A mRNA levels were determined 48 h after transfection of OTX2- or MITF-specific siRNA into ARPE-19 cells. Results are presented as the mean normalized expression±SD relative to cells transfected with non-specific siRNA (=100) from three independent transfections each performed in triplicate.

Because the RPE cell line D407 expresses MITF-A but lacks OTX2 and tyrosinase mRNAs (Figure 1), it was possible to test whether forced expression of OTX2 is sufficient to activate the *TYR* gene. After transfection into D407 cells and culture for 24 h (Figure 6A), OTX2 upregulated tyrosinase mRNA dose-dependently, from undetectable levels to more than a 100-fold increase. This suggests strongly that OTX2 is necessary for *TYR* expression in RPE cells.

### *TYR* expression in ARPE-19 cells is suppressed by specific small interfering RNAs against orthodenticle homeobox 2 and microphthalmia-associated transcription factor

The above experiments imply that control of the human *TYR* expression is mediated by OTX2 and MITFs. To confirm this, we reduced the expression of endogenous OTX2 and MITF with RNA silencing (Figure 6B). The *TYR*, *OTX2*, and *MITF-A* mRNA expression levels were decreased up to 63%, 61%, and 39%, respectively, with *OTX2* siRNA transfection. When *MITF* siRNA was used, the *TYR* and *MITF-A* mRNA levels were decreased up to 56% and 48%, respectively, whereas the *OTX2* levels were slightly increased. Despite the siRNAs’ moderate silencing efficiency, suppression of both transcription factors clearly reduced tyrosinase mRNA expression.

### Orthodenticle homeobox 2 and microphthalmia-associated transcription factor transcription factors interact physically

**Figure 7:**
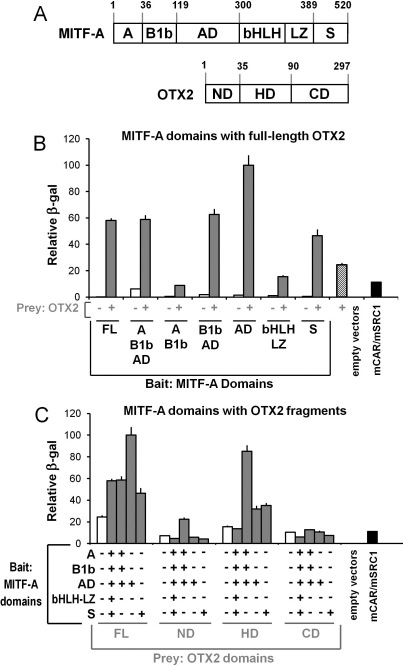
Protein–protein interactions occur between OTX2 and MITF-A. **A**: Full-length (FL) MITF-A and OTX2 and their known domains are shown with the corresponding amino acids as follows: MITF-A specific N-terminal (**A**), N-terminal region common in MITF-A and several other isoforms (B1b), activation domain (AD), basic helix–loop–helix leucine zipper (bHLH-LZ), and serine-rich C-terminal activation domain (S); OTX2 DNA-binding homeodomain (HD); and N- and C-terminal activation domains (ND, CD). **B**: Interactions of full-length OTX2 with various MITF-A domains (FL: aa1–520; A-B1b-AD: aa1–297; A-B1b: aa1–124; B1b-AD: aa36–297; AD: aa118–297; bHLH-LZ: aa295–405 or S: aa402–520) are shown by gray columns. Auto-activation of each “bait” MITF-A domain alone is indicated by white columns and for the full-length OTX2 “prey” construct by the hatched column. **C**: Interactions of selected MITF-A domains (FL, A-B1b-AD, AD, or S) with various OTX2 domains (FL: aa1−297; ND: aa1−37; HD: aa37−109 or CD: aa107−297) were assessed (gray columns) and compared with empty “bait” vectors (white columns). Assays were controlled with the yeast strain carrying empty “bait” and “prey” vectors or the positive control mCAR/SRC1 (black columns). Results are means±SEM relative to the maximal activation of the β-galactosidase reporter (=100) from three independent assays each performed in quadruplicate..

Because OTX2 can indirectly enhance *TYR* expression via the MITF sites (Figure 3), a potential interaction between OTX2 and MITF-A is implied. To study this interaction, we used the yeast two-hybrid assay and the modular structure of these two factors. MITF-A contains two distinct trans-activation domains (AD and S fragments) and a central DNA-binding domain encoded by the bHLH-LZ fragment [63]. OTX2 contains a DNA-binding homeodomain (HD) flanked by two trans-activation domains [62], termed here ND and CD (Figure 7A). In the yeast two-hybrid assays, we found high β-galactosidase reporter activities with the full-length MITF-A as bait and the full-length OTX2 as prey (Figure 7B, gray columns). Upon investigating various MITF-A bait fragments, the interaction required the presence of both activation domains (constructs containing domains AD or S) when the background activity of OTX2 alone (hatched columns) was taken into account. Among the MITF-A fragments, only the N-terminal A/B1b/AD domain displayed any substantial autoactivation of the reporter (Figure 7B, white columns). The MITF-A regions that showed reporter activity above the OTX2 prey alone were then individually tested for interactions with the full-length OTX2 and its subdomains. We saw generally modest reporter activities by MITF-A fragments with either N- and C-terminal trans-activation domains of OTX2. These activities were close to the autoactivation values of the OTX2 domains (Figure 7C, white columns). Instead, the DNA-binding homeodomain of OTX2 interacted with MITF-A activation domains AD and S, which supports the idea of physical interaction between the OTX2 and MITF-A transcription factors.

**Figure 8:**
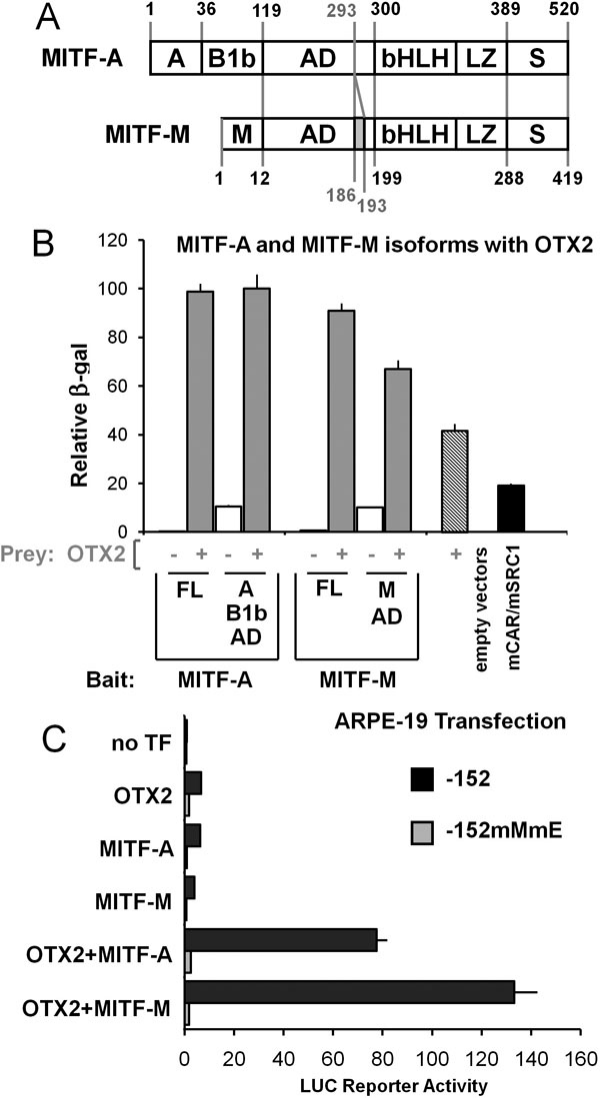
Effects of the MITF isoforms A and M are comparable. **A**: Full-length (FL) MITF isoforms A and M and their known domains are shown with the corresponding amino acids: MITF-A domains *A*, *B1b*, and *AD*, common domains *bHLH-LZ* and *S*, and the MITF-M specific N-terminal region (*M*). Additional six amino acids of the M isoform (aa187–192) are shown. **B**: Interactions of the full-length OTX2 and domains from the MITF isoform A (FL: aa1–520; A-B1b-AD: aa1–297) or M (FL: aa1–419; M-AD: aa1–196) were measured and are shown with gray columns. The level of MITF auto-activation is indicated by white columns. Assays were performed as in Figure 7 from three independent experiments each performed in quadruplicate. **C**: ARPE-19 cells were cotransfected with the proximal *TYR* promoter lacking any OTX2 sites but with intact (−152: black columns) or mutated MITF binding sites (−152mMmE: gray columns). Normalized luciferase activities are relative to *TYR* promoter construct −152 (=1) cotransfected with the empty expression vector pCR3 (=no TF). Data are means±SEM from two independent transfections each performed in triplicate.

Because ARPE-19 cells expressed *MITF-A* and *MITF-M* mRNAs (Figure 1) possessing common and isoform-specific domains (Figure 8A), we investigated whether the functions of the MITF isoforms were similar. Full-length MITF isoforms recruited OTX2 to a similar extent, while the N-terminal activation domain of MITF-M yielded a lower response than that of MITF-A (Figure 8B). In transient transfections with the construct −152 carrying the MITF but no OTX2 sites (Figure 8C), OTX2 and MITF-A activated the reporter by approximately 6.5-fold while MITF-M yielded a fourfold response. The synergism with OTX2 by the M isoform was about 1.7-fold greater than by MITF-A. The reporter with mutated MITF *M* and *E boxes* was not activated by any of the factors. Collectively, our data show that isoforms A and M can associate with OTX2 and synergize to activate the human *TYR* gene promoter.

## Discussion

In this study, we provide novel mechanisms for regulating the human *TYR* gene, and emphasize the role of OTX2 in the RPE. These findings might also contribute to understanding the role of OTX2 in regulating other MITF-controlled genes [64,65] in the RPE. Finally, we discuss the significance of the discovered functional SNP on the *TYR* expression and pathophysiology of the RPE.

An important role for OTX2 in human *TYR* regulation emerges from the following findings. First, the upregulation of *TYR* mRNA parallels the increase in OTX2 expression and precedes that of MITF isoforms, and OTX2- or MITF-specific siRNAs suppress *TYR* mRNA levels in ARPE-19 cells. Even though *OTX2* and *MITF* siRNAs were able to only partially downregulate their cognate targets by 50% to 60%, the *TYR* mRNA level decreased to the same extent (60%). This indicates a strong regulatory linkage between the human *TYR* promoter, *MITF*, and *OTX2*. Second, despite the presence of MITF-A and -H, *TYR* mRNA is expressed in D407 cells only after forced expression of OTX2. Moreover, the RPE-specific MITF-D was not expressed in D407 cells, and its role in *TYR* expression remains to be elucidated. Third, OTX2 can activate the *TYR* proximal promoter in ARPE-19 cells to much higher levels than MITF-A or MITF-M alone. The OTX2-dependent activation occurs via direct binding to the distal OTX2 sites and via indirect protein interaction at the proximal MITF sites.

This novel indirect activation is profoundly different from what Martinez-Morales and coworkers reported for the mouse *QNR71* promoter [49]. They demonstrated that intact OTX2 sites within the mouse *QNR71* promoter are obligatory for synergistic trans-activation by OTX2 and MITF, but they did not study the mouse *TYR* promoter in detail [49]. Our study shows clearly that OTX2 can activate the human *TYR* promoter, in the absence of any OTX2 binding elements, via MITF sites at the very proximal part of the promoter. Constructs lacking OTX2 sites 1 to 3 but harboring the M box had high levels of OTX2-dependent activity suggesting protein–protein interactions between factors binding to these closely spaced elements. The intricate pattern of nuclear protein binding at OTX2 site 3 is consistent with protein complexes that could represent OTX2, OTX2 dimers, and/or OTX2 in complex with MITF isoforms. Further, binding of OTX2 to site 3 was shown in supershift assays with specific antibodies. Next, we demonstrated a robust physical interaction between the full-length proteins that was mediated by the OTX2 homeodomain and the trans-activation domains of MITF. Our results contrast with the interaction detected in vitro between the recombinant full-length OTX2 and the bHLH domain of MITF [49]. However, both studies showed a direct interaction between OTX2 and MITF, and “bridging” factors are not necessary to explain the synergism in *trans*-activation or the EMSA band patterns. Redundancy in the interaction of OTX2 with MITF isoforms A and M also supports the finding that activation of the mouse *TYR* gene does not depend on the N-terminal structure of the MITF isoforms [37]. On a related note, both types of RPE cells displayed high activity when they were cotransfected with reporter constructs carrying *enhancer* and *TDE* elements. This suggests potential long-range interactions of OTX2 with MITF bound also at these distal locations [35,66].

Thus, a major role for OTX2 emerges in the regulation of *TYR* expression in human RPE cells. This view is supported by several knockout mouse models. First, mouse *Otx2*^−/−^ or *Otx2*^+/−^ embryos have abnormally developed eyes [39]. Second, the loss of *Otx2* disturbs the wingless (Wnt)/β-catenin pathway [41], which is necessary for the RPE phenotype, melanogenesis, and the response to oxidative stress [28]. Third, in *Otx*2^+/−^ mice, expression of *Mitf* and *TYR* genes is reduced and restricted to a few Otx2-positive cells [39]. Very recently, studies on conditional knockdown mice [67,68] have shown that elimination of OTX2 decreases melanogenesis and pigmentation of the RPE, culminating in degeneration of the retina. This correlates nicely with our findings on the role of OTX2 in regulating *TYR* and its relevance in vivo. Moreover, some OTX2 targets that control tyrosinase activity and sorting such as TRP-2 are also on the “human RPE signature gene list” [69].

We report here the presence of a functional SNP in the human *TYR* promoter that enforces the role of OTX2. Although the presence of tyrosinase activity in the adult RPE is controversial, the *TYR* gene is highly active in the developing eye [70]. Reduced melanogenesis in early life may have far-reaching functional effects on the aging RPE, and accordingly, fetal human RPE cells exhibited decreased reporter activity with the T-223C mutation. Certain RPE diseases such as age-related macular degeneration are linked to radiation or oxidative damage and, thus, inversely correlated with protection by melanin [6]. The prevalence of age-related macular degeneration is associated with RPE depigmentation [71,72] and less frequent among persons of African descent [73–75]. These phenotypes are therefore consistent with the OTX2 and the polymorphic *site 3* having a significant role in tyrosinase and melanin production in the RPE. The view that common polymorphisms associated with common traits are likely to be located in regulatory elements of the gene [75], as exemplified by the vitamin K epoxide reductase complex 1 (VKORC1) gene [76], are also in line with our conclusions. Therefore, it would be interesting to study the association of this SNP with levels of RPE melanin or with other RPE diseases in human subjects.

In addition, due to the expression of OTX2 in the central nervous tissue [42], and the presence of tyrosinase in the substantia nigra and other parts of the brain [77], the question of the role of OTX2 and the polymorphic site 3 in susceptibility to Parkinson disease arises. Depigmentation and/or loss of pigmented cells in the substantia nigra and the locus ceruleus are typical findings in Parkinson disease [78–81], and epidemiological studies have shown variation among ethnic groups in the prevalence of Parkinson disease [82]. However, the regulation of neuromelanin biosynthesis in pigmented neurons is unclear and requires further study. In conclusion, our findings clearly warrant further studies on the interplay of OTX2 and MITF and on the genetic variation associated with human *TYR* gene regulation in the retina and other pigmented cells.

## Appendix 1.

Supplemental methods. Cloning of the human tyrosinase promoter and construction of promoter deletions; and Preparation of nuclear extracts and EMSA reactions. To access the data, click or select the words “Appendix 1.” This will initiate the download of a compressed (pdf) archive that contains the file. Complementary antisense primers for mutagenesis are not shown. Mutated nucleotides are shown with capital letters and the positions of binding sites are underlined.

## Appendix 2.

Sense primers for the site-directed mutagenesis of MITF and OTX2 transcription factor binding sites. To access the data, click or select the words “Appendix 2.” This will initiate the download of a compressed (pdf) archive that contains the file. Complementary antisense primers for mutagenesis are not shown. Mutated nucleotides are shown with capital letters and the positions of binding sites are underlined.

## Appendix 3.

Sense oligonucleotides for the electrophoretic mobility shift assays. To access the data, click or select the words “Appendix 3.” This will initiate the download of a compressed (pdf) archive that contains the file. The positions of the binding elements are underlined and mutated nucleotides are in capital letters. For each oligonucleotide, four unspecific nucleotides at the 5′-end were designed for fill-in labeling with [α-^32^P] dCTP. Complementary antisense sequences are not shown.

## Appendix 4.

OTX2 activates the tyrosinase promoter through the proximal region at −462 to −152. To access the data, click or select the words “Appendix 4.” This will initiate the download of a compressed (pdf) archive that contains the file. Luc-reporter constructs containing the human tyrosinase promoter −2525 with enhancer element −9860 to −8741 (=Enh./-2525), deletions −2525, −1995, −462, −152 and promoterless pGL3-Basic were cotransfected into D407 RPE cells with MITF-A, OTX2 or empty expression vector (pCR3). Normalized luciferase activities are relative to tyrosinase promoter construct −152 (=1) co-transfected with empty expression vector (pCR3). Data are means±SEM from two independent transfections performed in triplicate.

## Appendix 5.

In the RPE, OTX2 activates the tyrosinase promoter in the absence of MITF binding elements. To access the data, click or select the words “Appendix 5.” This will initiate the download of a compressed (pdf) archive that contains the file. Luc-reporter constructs containing deletions of the MITF binding elements at positions −104 to −99 (mM=deletion of the M-box) and −12 to −7 (mE=deletion of the E-box) in the human tyrosinase promoters −152 or −462 were co-transfected into D407 cells with MITF-A, OTX2, MITF-A/OTX2 factors or with empty expression vector (pCR3). Normalized luciferase activities are relative to tyrosinase promoter construct −152 (=1) co-transfected with empty expression vector pCR3 (=vector only). Data are means±SEM from two independent transfections performed in triplicate.

## Appendix 6.

OTX2 activates the tyrosinase promoter through the specific OTX2 binding elements 1 (−361 to −356), 2 (−354 to −349) and 3 (−226 to −221) and through the protein–protein interactions with MITF. To access the data, click or select the words “Appendix 6.” This will initiate the download of a compressed (pdf) archive that contains the file. Luc-reporter constructs containing deletions of the OTX2 binding elements 1, 2 and/or 3 (renamed as m1, m2 and m3) in the human tyrosinase promoter −462 were co-transfected into D407 cells with MITF-A, OTX2, MITF-A/OTX2 factors or with empty expression vector (pCR3). Normalized luciferase activities are relative to tyrosinase promoter construct −152 (=1) co-transfected with empty expression vector pCR3 (not shown). Data are means±SEM from two independent transfections performed in triplicate.

## Appendix 7.

SNP (rs4547091) in OTX2 binding element 3 (−226 to −221) decrease the activity of the human tyrosinase promoter dramatically in the presence of OTX2 and MITF-A. To access the data, click or select the words “Appendix 7.” This will initiate the download of a compressed (pdf) archive that contains the file. Lucreporter construct containing the tyrosinase promoter fragments Enh./-2525, Enh./-2525SNP, −462, −462SNP or −462m3 were co-transfected into D407 RPE cells with MITF-A, OTX2, MITF-A/OTX2 factors or with empty expression vector (pCR3). Normalized luciferase activities are relative to tyrosinase promoter construct −152 (=1) co-transfected with empty expression vector pCR3 (not shown). Data are means±SEM from two independent transfections performed in triplicate.

## Appendix 8.

Nuclear protein binding to proximal MITF binding elements in the human *TYR* promoter. Nuclear protein binding to M-box and E-box binding elements was studied by using double-stranded DNA probes and nuclear extracts from ARPE-19 cells cultured for 14 days. To access the data, click or select the words “Appendix 8.” This will initiate the download of a compressed (pdf) archive that contains the file. Top panel, labeled M-box probe was competed with a sixfold excess of unlabelled probes for unrelated GAL4 (lane 2), wild-type M-box (lane 3), mutated M-box (lane 4) or wild-type OTX2 site 3 (lane 5). Bottom panel, labeled E-box probe was competed with a sixfold excess of unlabeled probes for unrelated GAL4 (lane 2), wild-type E-box (lane 3), mutated E-box (lane 4) or wild-type OTX2 site 3 (lane 5). Specific DNA–protein complexes are indicated with symbols A, A1 or A2 while non-specific complexes are indicated with B. Binding reactions in the absence of competitor are on lanes 1.
